# Career Compromise, Perceived Organizational Support, and Professional Identity in Nurses: A Latent Profile Analysis and Mediation Study

**DOI:** 10.1155/jonm/3901921

**Published:** 2026-10-26

**Authors:** Fang Zhao, Jing Jiang, Yifang Zhang, Linyan Dong, Xinyue Liu, Junmei Zhang

**Affiliations:** ^1^ Department of Nursing Henan Provincial Chest Hospital Zhengzhou University Zhengzhou 450008 Henan China zzu.edu.cn; ^2^ School of Nursing and Health Zhengzhou University Zhengzhou 450001 Henan China zzu.edu.cn; ^3^ Health Care Office Henan Provincial People’s Hospital Zhengzhou 450003 Henan China hnsrmyy.net

**Keywords:** career compromise (CC), latent profile analysis, mediating effect, nurse, perceived organizational support (POS), professional identity (PI)

## Abstract

**Objective:**

To characterize unobserved patterns of nurses’ professional identity (PI), identify factors related to profile membership, and determine whether perceived organizational support (POS) statistically accounts for part of the association between career compromise (CC) and PI.

**Methods:**

A cross‐sectional electronic questionnaire was completed by 2288 nurses recruited from 10 hospitals in Henan Province, China, between August and November 2025. The assessment tools utilized comprised a general information questionnaire, the Career Compromise Scale, the Perceived Organizational Support Scale, and the Nurses’ Professional Identity Scale. Latent profile analysis (LPA) was employed to derive the PI subgroups. Group differences and profile correlates were evaluated with univariate analyses and multinomial logistic regression, respectively. The assessment of the indirect connection between CC and PI via POS was carried out utilizing Pearson correlation and SEM.

**Results:**

The classification of nurses′ PI resulted in three latent profiles: low‐PI (30.4%), moderate‐PI (43.4%), and high‐PI (26.2%). Holding a position in nursing management and earning a higher income were associated with an increased likelihood of belonging to the moderate‐ and high‐patient impact groups. Conversely, the likelihood of nurses in acute/critical care units or emergency departments being part of the moderate‐ and high‐PI groups was lower. After accounting for various factors, having more than 10 night shifts monthly was not significantly related to high‐PI group membership. According to the SEM, CC was positively linked to POS (*β* = 0.901, *p* < 0.01) and PI (*β* = 0.463, *p* < 0.01), and POS had a positive link with PI (*β* = 0.382, *p* < 0.01); the standardized indirect effect amounted to 0.344.

**Conclusion:**

Nurses’ PI was represented by three profiles. Membership varied by income, management role, department, gender, hospital level, and hospital type, whereas monthly night‐shift frequency did not independently differentiate the high‐PI profile after covariate adjustment. POS also accounted for a significant portion of the positive CC–PI association.

## 1. Introduction

Professional identity (PI) in nursing encompasses how nurses understand their professional role, attach value and emotion to it, and orient themselves within the profession [1]. The International Society for Professional Identity in Nursing (ISPIN) defines PI as a self‐perception that is cultivated in relation to others within the field. This self‐perception is significantly influenced by the established norms, values, and characteristics inherent to the discipline of nursing. As a result, PI plays a crucial role in shaping thoughts and behaviors that are specific to nursing practice. By understanding PI in this context, nurses can better recognize how their PI develops and influences their interactions and decision‐making within the healthcare environment [1]. Stronger PI has been linked to career well‐being, engagement, resilience, intention to remain, and safe, high‐quality care [1–4]. PI is nevertheless heterogeneous. A Shanghai study identified sizeable low‐PI and indifferent subgroups among newly employed nurses [2], while a different profile structure has been reported among undergraduate nursing interns [5]. A person‐centered approach can reveal such between‐nurse variation that an overall score may conceal.

Career compromise (CC) is the result of practical limitations causing individuals to alter or lower their expectations for occupational interests, prestige, person‐career alignment, or advancement prospects [6–8]. Negative consequences, like negative affect, pulling away from objectives, burnout, lower satisfaction at work, and the desire to resign, have generally been accompanied by more compromise [6, 8, 9]. However, its link to nurses′ PI remains undetermined and could be influenced by work environment and the measurement of CC.

Employees perceive organizational support (POS) as their overall belief that the organization appreciates their contributions and cares about their well‐being [10, 11]. According to Organizational Support Theory and Social Exchange Theory, it is probable that nurses will increase their engagement, commitment, and sense of identification with their professional roles in response to the emotional and practical support they perceive [10, 11]. The Conservation of Resources Theory suggests that when facing professional challenges or stress at work, individuals can maintain their psychological resources through support provided by their organization [12]. Therefore, POS might serve as a connecting factor between career adjustment experiences and PI, rather than just being a contextual feature.

Existing evidence does not yet connect CC, POS, and PI coherently. PI profiles have been examined in newly employed nurses and interns [2, 5], but evidence from large samples of practicing nurses remains limited. In the nursing profession, CC has been associated with burnout, levels of job satisfaction, and the desire to resign from their positions [9], yet its association with PI—particularly when compromise may reflect both constraint and pragmatic adjustment—is unclear. POS has separately been associated with nursing work outcomes and PI [10, 11, 13], but the three constructs have rarely been evaluated together. Because latent profile analysis (LPA) detects unobserved groups from shared response patterns, it can characterize heterogeneity in PI [14]; pairing it with SEM permits simultaneous examination of PI profiles and the associations linking CC, POS, and PI.

Drawing on Social Exchange Theory, Organizational Support Theory, and Conservation of Resources Theory, we expected heterogeneous PI profiles among nurses and a partial indirect CC–PI association through POS.

## 2. Methods

### 2.1. Research Design and Data Collection

This cross‐sectional investigation was conducted and recorded in accordance with the STROBE guidelines for observational research in epidemiology [15]. Nurses working in 10 hospitals located in Henan Province, China, were selected through a convenience sampling approach. Data collection was conducted electronically via the Wenjuanxing platform from August through November 2025. Trained coordinators at each hospital distributed a questionnaire link or QR code to eligible nurses. Prior to completing the questionnaire, participants reviewed a standardized information sheet that explained the study’s purpose, the voluntary aspect of participation, and ensured anonymity, estimated completion time, confidentiality of data, and the ability to withdraw prior to submission. Only attendees who provided informed consent were allowed to proceed. The purpose of this study was to accomplish the following analytical goals: (1) to determine hidden patterns of PI among nurses; (2) to examine the associations between sociodemographic and work‐related factors and profile membership; and (3) to investigate the role of POS in mediating the link between CC and PI.

The requirements of both LPA and multinomial logistic regression were considered in sample planning. For LPA, the required sample varies with the number of profiles, class separation, and model complexity; simulation studies show that larger samples improve classification and the power of likelihood‐ratio tests [16, 17]. Larger samples likewise provide more precise SEM parameters and mediation estimates [18]. We therefore sought more than 2000 valid responses. Before formal distribution, the research team conducted a technical and readability check of the electronic questionnaire to verify item logic, page display, and estimated completion time; data from this pretest were not included in the formal analysis. Of 2500 questionnaires distributed, 2350 were returned (94.00%). Quality screening removed 62 responses with patterned answers, clear logical inconsistencies, or failure on prespecified checks, leaving 2288 questionnaires for analysis (97.36% of returned questionnaires). Mandatory fields prevented item‐level missingness in the analytic dataset. Inclusion criteria were as follows: (1) possessing a current nursing license, being registered, and actively working; (2) having been employed in a clinical frontline role for at least 1 year; (3) not presently using medication for any mental health or psychological conditions; and (4) voluntarily agreeing to participate. Exclusion criteria were as follows: (1) interns, visiting students, trainees, and other individuals who were not employed as registered nurses by the participating hospitals; and (2) being on extended leave, participating in training, or not on duty during the survey duration.

### 2.2. Research Tools

#### 2.2.1. General Information Questionnaire

A custom‐made general information questionnaire was created to gather the following details: gender, age, hospital type, hospital level, employment type, department, relationship status, count of offspring, educational level, years in the field, professional role, whether holding a nursing management position, monthly night shift (0, 1–5, 6–10 times or > 10 times), personal monthly income (≤ 5000 yuan, > 5000–10,000 yuan, > 10,000–15,000 yuan, or > 15,000 yuan).

#### 2.2.2. The Career Compromise Scale

The Career Compromise Scale was created by a team of Chinese researchers led by Weng et al. [8]. This scale consisted of three dimensions: sacrifice potential for development (4 items), compromise on career match (4 items), and compromise on social expectations (4 items). Each item was rated on a 5‐point Likert scale (1–5 points correspond to *Strongly Disagree* and *Strongly Agree*). The final score ranged from 12 to 60; an increased score reflects a higher level of CC. The Cronbach’s *α* coefficient for the scale in this study was 0.959, demonstrating strong reliability.

#### 2.2.3. The Perceived Organizational Support Scale

The scale measuring POS for nurses was created by Chinese researcher Chen and later revised by Zuo [19]. Ten items assess emotional support, and three assess instrumental support. Each item is rated from 1 (*Very inconsistent*) to 5 (*Very consistent*), producing a total score of 13–65. Higher totals indicate stronger POS. In this study, the Cronbach′*α* coefficient of this scale was 0.980.

#### 2.2.4. The Nurse Professional Identity Scale

The Nurse Professional Identity Scale was developed by Chinese researchers Liu et al. [20]. Its 30 items cover professional cognitive assessment (9 items), professional social support (6 items), professional social skills (6 items), coping with career setbacks (6 items), and professional self‐reflection (3 items). Responses range from 1 (*Very inconsistent*) to 5 (*Very consistent*), yielding a total score of 30–150. A higher score indicates a higher level of nurses’ PI. Based on the total score, participants can be categorized into four predefined PI levels: low (30–60), medium‐low (61–90), moderate (91–120), and high (121–150). In this study, the Cronbach’s *α* coefficient of this scale was 0.983.

### 2.3. Quality Control and Ethical Considerations

Quality assurance measures were applied during the questionnaire setup and after the survey closed. Wenjuanxing ensured that each IP address could only submit once, and all items required responses. Three investigators, trained in medical research methods, reviewed the records in two stages. They excluded responses that demonstrated straight‐lining, alternating extremes (such as ′1, 5, 1, 5…′), any evident logical contradictions, or completion. Before data were collected, the participants received comprehensive information about the study’s objectives, methods, and their rights, and they gave their electronic informed consent. To ensure privacy protection, the research employed a confidential survey and did not collect any personal identifiers like names or ID numbers. This study received approval from the Ethics Review Committee at Zhengzhou University (Approval No.: ZDHL20250501).

### 2.4. Statistical Methods

Mplus 8.3 software was used to perform a LPA. LPA groups participants whose patterns across continuous indicators are similar but not directly observed [14]. Models with different class counts were compared using AIC, BIC, aBIC, entropy, and the likelihood ratio test using bootstrapping (BLRT). Smaller AIC, BIC, and aBIC values favor a model, whereas a significant BLRT indicates improvement over a solution with one fewer class. Entropy approaches 1 as classification becomes clearer [14]. Additionally, class size, posterior classification probability, theoretical interpretability, and clinical utility were also considered in the model selection. Consistent with LPA recommendations, solutions containing very small classes (e.g., any class with a minimum probability below approximately 10%) were interpreted with caution, as such solutions may reflect over‐extraction and may have limited practical utility in clinical management [14].

All statistical analyses utilized RStudio, specifically Version 4.2.2. Frequencies (*N*) and percentages (%) were used to summarize categorical variables, while continuous variables were described using the mean ± standard deviation (SD).

To examine differences among the three PI profiles, one‐way ANOVA was employed for variables that change continuously, Chi‐square tests were applied to categorical variables. Variables with a *p* value under 0.05 in univariate analyses were included in a multinomial logistic regression model, with the low‐PI group serving as the reference category. This model separately compared the moderate‐PI and high‐PI groups to the low‐PI group, offering odds ratios (ORs) along with 95% confidence intervals (CIs). Table 1 displays the assignment and reference categories for each independent variable.

**TABLE 1:** Variable assignment for multinomial logistic regression.

Variable	Assignment/coding	Reference category
Dependent variable: PI profile	Low‐PI = reference; Moderate‐PI = comparison 1; High‐PI = comparison 2	Low‐PI group
Gender	Male = 0; Female = 1	Male
Hospital type	General hospital = 0; Specialized hospital = 1	General hospital
Hospital level	Tertiary hospital = 0; Secondary hospital = 1	Tertiary hospital
Department	Clinical specialty departments; acute/critical care units and emergency departments; medical technology and platform; nursing administration and integrated services	Clinical specialty departments
Nursing management position	No = 0; Yes = 1	No
Monthly night shifts	0; 1–5; 6–10; > 10 times/month	0 times/month
Monthly income	≤ 5000; > 5000–10,000; > 10,000–15,000; > 15,000 yuan	≤ 5000 yuan

We calculated Pearson correlations among CC, POS, and PI. AMOS 26.0 was then used to fit an SEM in which POS represented the proposed indirect link between CC and PI. The model’s fit was assessed using the *χ* ^2^/df, RMSEA, NFI, TLI, IFI, and CFI. Acceptable fit was defined as χ^2^/df < 3, RMSEA < 0.08, and NFI, TLI, IFI, and CFI > 0.90 [21]. Given the sensitivity of the χ^2^ statistic to large sample sizes, the Bollen–Stine bootstrap test with 2000 bootstrap samples was additionally used to assess exact model fit [22, 23].

## 3. Results

### 3.1. General Information

This study included a total of 2288 nurses. The average overall age was (33.18 ± 6.46) years, with females accounting for 85.71%. In terms of hospital types, specialized hospitals and general hospitals accounted for 45.02% and 54.98%, respectively. The majority of hospitals were classified as tertiary (92.70%). The most common employment type was personnel agency (73.34%), and most of the nurses were affiliated with clinical specialties (61.01%). The majority of the educational qualifications were bachelor’s degree or above (93.66%), and the most common professional title was senior nurse (56.03%). The majority of nurses did not hold nursing management positions (93.66%). The number of night shifts per month was mainly 6–10 (46.28%), and the most common personal monthly income fell within the range of 5000–10,000 yuan (39.90%). More details are presented in Table 2.

**TABLE 2:** Univariate analysis of sociodemographic and work‐related characteristics of different PI groups (*N* = 2288).

Variables	Total (*n* = 2288)	Low‐PI group (*n* = 692)	Moderate‐PI group (*n* = 995)	High‐PI group (*n* = 601)	Statistic	*p*
Age, mean ± SD	33.18 ± 6.46	32.87 ± 6.00	33.40 ± 6.76	33.17 ± 6.45	*F* = 1.40	0.246
Gender, *n* (%)					*χ* ^2^ = 9.30	0.010
Male	327 (14.29)	86 (12.43)	133 (13.37)	108 (17.97)		
Female	1961 (85.71)	606 (87.57)	862 (86.63)	493 (82.03)		
Hospital type, *n* (%)					*χ* ^2^ = 24.32	< 0.001
Specialized hospital	1030 (45.02)	286 (41.33)	422 (42.41)	322 (53.58)		
General hospital	1258 (54.98)	406 (58.67)	573 (57.59)	279 (46.42)		
Hospital level, *n* (%)					*χ* ^2^ = 26.09	< 0.001
Secondary hospital	167 (7.30)	73 (10.55)	75 (7.54)	19 (3.16)		
Tertiary hospital	2121 (92.70)	619 (89.45)	920 (92.46)	582 (96.84)		
Employment type, *n* (%)					*χ* ^2^ = 5.72	0.221
Employed through a Personnel Agency	1678 (73.34)	524 (75.72)	731 (73.47)	423 (70.38)		
Contract personnel	465 (20.32)	131 (18.93)	196 (19.70)	138 (22.96)		
Permanent staff	145 (6.34)	37 (5.35)	68 (6.83)	40 (6.66)		
Department, *n* (%)					*χ* ^2^ = 17.13	0.009
Nursing administration and integrated services	63 (2.75)	18 (2.60)	32 (3.22)	13 (2.16)		
Medical technology and platform departments	457 (19.97)	139 (20.09)	185 (18.59)	133 (22.13)		
Clinical specialty departments	1396 (61.01)	399 (57.66)	615 (61.81)	382 (63.56)		
Acute/critical care units and emergency departments	372 (16.26)	136 (19.65)	163 (16.38)	73 (12.15)		
Marital status, *n* (%)					*χ* ^2^ = 1.99	0.370
Unmarried	631 (27.58)	185 (26.73)	267 (26.83)	179 (29.78)		
Married	1657 (72.42)	507 (73.27)	728 (73.17)	422 (70.22)		
Number of children, *n* (%)					*χ* ^2^ = 3.17	0.788
0	778 (34.00)	241 (34.83)	327 (32.86)	210 (34.94)		
1	768 (33.57)	224 (32.37)	351 (35.28)	193 (32.11)		
2	709 (30.99)	215 (31.07)	303 (30.45)	191 (31.78)		
≥ 3	33 (1.44)	12 (1.73)	14 (1.41)	7 (1.16)		
Education level, *n* (%)					*χ* ^2^ = 3.95	0.139
College degree and below	145 (6.34)	52 (7.51)	64 (6.43)	29 (4.83)		
Bachelor’s degree or above	2143 (93.66)	640 (92.49)	931 (93.57)	572 (95.17)		
Years of service, *n* (%)					*χ* ^2^ = 10.36	0.241
≤ 5 years	504 (22.03)	154 (22.25)	209 (21.01)	141 (23.46)		
> 5–10 years	530 (23.16)	162 (23.41)	243 (24.42)	125 (20.80)		
> 10–15 years	760 (33.22)	242 (34.97)	317 (31.86)	201 (33.44)		
> 15–20 years	305 (13.33)	92 (13.29)	132 (13.27)	81 (13.48)		
> 20 years	189 (8.26)	42 (6.07)	94 (9.45)	53 (8.82)		
Professional title, *n* (%)					*χ* ^2^ = 9.46	0.150
Registered nurse	274 (11.98)	84 (12.14)	110 (11.06)	80 (13.31)		
Staff nurse	619 (27.05)	184 (26.59)	276 (27.74)	159 (26.46)		
Deputy chief nurse and above	113 (4.94)	26 (3.76)	46 (4.62)	41 (6.82)		
Senior nurse	1282 (56.03)	398 (57.51)	563 (56.58)	321 (53.41)		
Nursing management, *n* (%)					*χ* ^2^ = 27.25	< 0.001
No	2143 (93.66)	675 (97.54)	922 (92.66)	546 (90.85)		
Yes	145 (6.34)	17 (2.46)	73 (7.34)	55 (9.15)		
Monthly night shift, *n* (%)					*χ* ^2^ = 18.19	0.006
0	522 (22.81)	147 (21.24)	230 (23.12)	145 (24.13)		
1–5	565 (24.69)	147 (21.24)	251 (25.23)	167 (27.79)		
6–10	1059 (46.28)	352 (50.87)	442 (44.42)	265 (44.09)		
> 10	142 (6.21)	46 (6.65)	72 (7.24)	24 (3.99)		
Monthly income, *n* (%)					*χ* ^2^ = 75.21	< 0.001
≤ 5000 yuan	614 (26.84)	257 (37.14)	260 (26.13)	97 (16.14)		
> 5000–10,000 yuan	913 (39.90)	241 (34.83)	409 (41.11)	263 (43.76)		
> 10,000–15,000 yuan	604 (26.40)	157 (22.69)	254 (25.53)	193 (32.11)		
> 15,000 yuan	157 (6.86)	37 (5.35)	72 (7.24)	48 (7.99)		

### 3.2. LPA of Nurses’ PI

Models with 1–5 latent profiles were sequentially fitted using the five dimensions of the Professional Identity Scale as observable variables. AIC, BIC, and aBIC declined from the one‐profile through the three‐profile model (Table 3). The three‐profile solution exhibited an entropy value of 0.965 and a BLRT that was significant (*p* = 0.01). Despite the fact that the AIC and BIC values for the 4‐class and 5‐class models decreased further, their smallest class probabilities were 9.3% and 8.7%, respectively, both below the prespecified 10% interpretability threshold. Given the small groups in the four‐ and five‐profile solutions, the three‐profile model offered a clearer balance of fit, class size, and substantive interpretation [14]. The final class probabilities were 30.4%, 43.4%, and 26.2%, respectively.

**TABLE 3:** Latent profile analysis of PI.

Classes	AIC	BIC	SABIC	Entropy	BLRT val	BLRT p	Class probability
1	32480.31	32537.67	32505.9	1	NA	NA	NA
2	25010.06	25101.82	25050.99	0.933	7482.256	0.01	0.679/0.321
3	18303.43	18429.6	18359.71	0.965	6718.64	0.01	0.304/0.434/0.262
4	17708.88	17869.47	17780.51	0.944	1195.703	0.01	0.093/0.294/0.395/0.217
5	16484.37	16679.37	16571.35	0.926	1782.525	0.01	0.338/0.212/0.175/0.188/0.087

Based on the score profiles of the three classes across the dimensions of PI (Figure 1), Class 3 was named the Low‐PI Group (30.4%), which showed low scores in all dimensions. Class 1 was named the Moderate‐PI group (43.4%), with scores that were moderate across all dimensions. Class 2 was named the High‐PI group (26.2%), exhibiting the highest scores across all dimensions.

**FIGURE 1:**
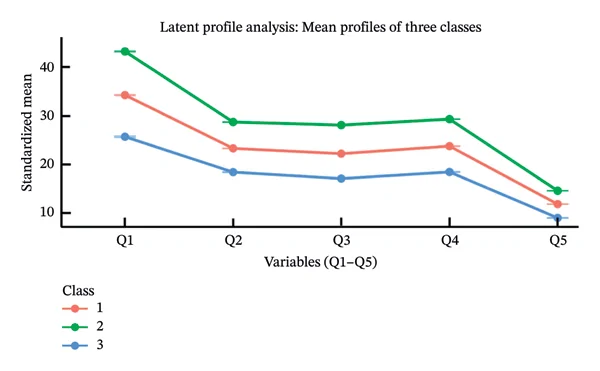
Latent profile characteristics of nurses’ PI. Note: Q1 represents professional cognitive assessment; Q2 represents professional social support; Q3 represents professional social skills; Q4 represents career setback coping; Q5 represents professional self‐reflection.

### 3.3. Univariate Comparison Among Different PI Profiles

According to the univariate analysis, significant statistical differences were found among the three PI groups regarding gender, type of hospital, hospital level, department, nursing management roles, monthly night shifts, and personal monthly income (all *p* < 0.05). Statistical analysis revealed no significant differences in age, employment type, spousal status, number of kids, level of education, years in the workforce, and career designation (all *p* > 0.05). The results of the univariate comparisons are presented in Table 2.

### 3.4. Analysis Using Multivariate Logistic Regression on Factors Affecting Latent Profiles of Nurses’ PI

The multinomial model included the seven variables associated with profiles at *p* < 0.05 in the univariate analyses: gender, hospital type, hospital level, department, nursing management status, monthly night shifts, and income. Table 1 shows variable assignments and reference categories. Using the low‐PI group as a baseline, nurses with a higher monthly income were more likely to be in the moderate‐PI group (> 5000–10,000 yuan: OR = 1.71, 95% CI: 1.30–2.23; > 10,000–15,000 yuan: OR = 1.74, 95% CI: 1.26–2.41; > 15,000 yuan: OR = 1.92, 95% CI: 1.19–3.08) and the high‐PI group (> 5000–10,000 yuan: OR = 2.29, 95% CI: 1.65–3.19; > 10,000–15,000 yuan: OR = 2.46, 95% CI: 1.68–3.60; > 15,000 yuan: OR = 2.29, 95% CI: 1.33–3.93). Being in a nursing management role was linked to higher chances of being part of the moderate‐PI group (OR = 2.80, 95% CI: 1.60–4.91) or the high‐PI group (OR = 4.08, 95% CI: 2.26–7.38). Compared to their counterparts in clinical specialty departments, nurses in acute/critical care and emergency departments had reduced chances of being in the moderate‐PI group (OR = 0.71, 95% CI: 0.53–0.96) or the high‐PI group (OR = 0.50, 95% CI: 0.35–0.72). Female nurses (OR = 0.47, 95% CI: 0.33–0.67) and nurses working in secondary hospitals (OR = 0.46, 95% CI: 0.26–0.82) were less likely to belong to the high‐PI group. Nurses working in specialized hospitals had greater odds of high‐PI group membership (OR = 1.33, 95% CI: 1.01–1.77), but this was not observed for the moderate‐PI group. For nurses working more than 10 night shifts per month, the adjusted estimate for high‐PI membership failed to achieve statistical significance (OR = 0.59, 95% CI: 0.33–1.04). The adjusted odds ratios and 95% confidence intervals are shown in Figure 2.

**FIGURE 2:**
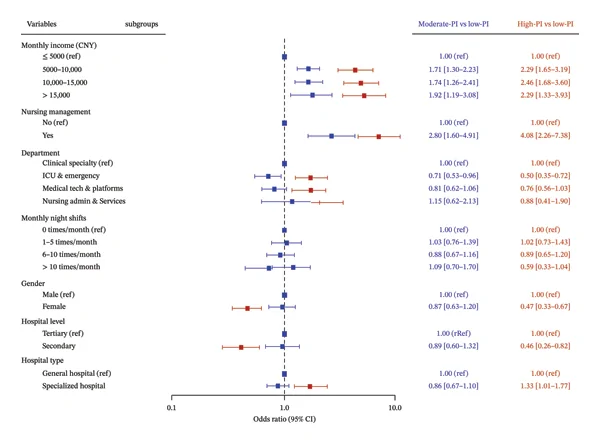
Forest plot of multinomial logistic regression results for PI profile membership.

### 3.5. Associations and Mediating Role Among CC, POS, and PI

#### 3.5.1. Correlation Analysis

Each CC and POS dimension correlated positively with PI (*r* = 0.661–0.762, all *p* < 0.01). The largest correlations with PI were observed for emotional support (*r* = 0.762) and compromise on social expectations (*r* = 0.750). Positive correlations of significance were also detected within and between the CC and POS dimensions (*r* = 0.708–0.881, *p* < 0.01). The correlation coefficients are presented in Table 4.

**TABLE 4:** Correlation analysis of CC, POS, and PI.

Correlation coefficient (*r*)	Compromise on development opportunities	Compromise on career fit	Compromise on social expectations	Emotional support	Instrumental support	Nurse PI
Compromise on development opportunities	1					
Compromise on career fit	0.827^∗∗^	1				
Compromise on social expectations	0.756^∗∗^	0.839^∗∗^	1			
Emotional support	0.745^∗∗^	0.781^∗∗^	0.836^∗∗^	1		
Instrumental support	0.708^∗∗^	0.744^∗∗^	0.744^∗∗^	0.881^∗∗^	1	
Nurse PI	0.661^∗∗^	0.739^∗∗^	0.750^∗∗^	0.762^∗∗^	0.728^∗∗^	1

*Note:* The double asterisks (^∗∗^) indicate statistical significance at *p* < 0.01 (two‐tailed).

#### 3.5.2. Model Fitting Results

In the SEM, CC was identified as the exogenous construct, POS served as the mediator, and PI was the outcome, as shown in Figure 3. The parameters were estimated using maximum likelihood. A significant result was observed in the initial chi‐square test because of the large sample size (*N* = 2288), with a *χ* ^2^/df of 27.040. Thus, we utilized the Bollen–Stine method with 2000 bootstrap samples. Postcorrection, all fit indices adhered to the suggested criteria (*χ* ^2^/df = 1.63, RMSEA = 0.02, CFI = 1.00, etc.; refer to Table 5), demonstrating that the hypothesized association model aligned well with the data and could be utilized for path analysis.

**FIGURE 3:**
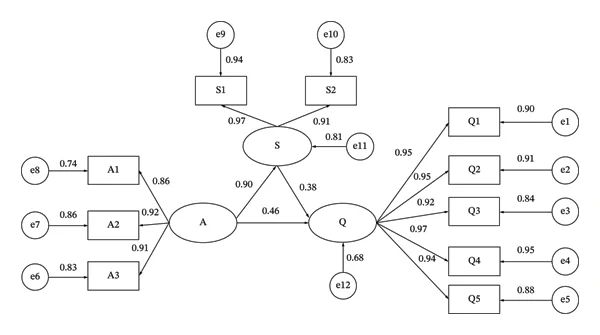
Structural equation model of CC, POS, and PI.

**TABLE 5:** Fit indices for the structural equation model.

Fit indices	Preliminary fitting (ML)	Bollen–Stine P‐corrected fit values	Ideal criteria
*χ* ^2^/df	27.040	1.63	< 3.0
RMSEA	0.107	0.02	< 0.08
GFI	0.973	1.00	> 0.90
AGFI	0.962	1.00	> 0.90
NFI	0.973	1.00	> 0.90
TLI	0.964	1.00	> 0.90
IFI	0.974	1.00	> 0.90
CFI	0.974	1.00	> 0.90

*Note:* ML, maximum likelihood estimation method.

#### 3.5.3. The Association Between CC and PI: The Mediating Role of POS

CC had positive standardized paths to POS (*β* = 0.901, *p* < 0.01) and PI (*β* = 0.463, *p* < 0.01); the POS–PI path was also positive (*β* = 0.382, *p* < 0.01). The product of the two component paths was 0.344 (0.901 × 0.382), consistent with partial mediation. The specific path coefficients are presented in Table 6 and Figure 3. Table 6 reports the nonstandardized estimates, standard errors, critical ratios, *p* values, and standardized estimates.

**TABLE 6:** Path regression coefficients of the structural equation model.

Parameter	Nonstandardized estimates	Standard error	CR value	*p* value	Standardized reference estimate
CC ⟶ POS	2.362	0.037	64.181	< 0.01	0.901
POS ⟶ PI	0.319	0.032	9.887	< 0.01	0.382
CC ⟶ PI	1.016	0.084	12.038	< 0.01	0.463

*Note:* Hidden variables are shown as ellipses, and observed variables are depicted as rectangles. The numbers on single‐headed arrows indicate standardized path coefficients, and the numbers next to observed variables indicate factor loadings. e1–e12 represent residual terms. A (CC) is evaluated by A1 (sacrificing development opportunities), A2 (sacrificing career alignment), and A3 (sacrificing social expectations). S (POS) is assessed through S1 (emotional support) and S2 (instrumental support).Q (PI) is evaluated through Q1 (assessment of professional cognition), Q2 (support from professional social networks), Q3 (skills in professional social interactions), Q4 (coping with career setbacks), and Q5 (self‐reflection in a professional context).

## 4. Discussion

This study integrated LPA, multinomial logistic regression, and SEM to examine the heterogeneity of nurses’ PI and the associations among CC, POS, and PI. The main findings were as follows: (1) Nurses’ PI comprised three distinct latent profiles: low‐PI, moderate‐PI, and high‐PI groups; (2) Profile membership was associated with income, nursing management position, department, gender, hospital level, and hospital type, whereas night‐shift frequency was not a robust predictor of high‐PI membership after adjustment; (3) The path pattern was consistent with an indirect association between CC and PI via POS. The following discussion focuses on the substantive implications of these findings and avoids causal overinterpretation because of the cross‐sectional design.

### 4.1. Heterogeneity in Nurses’ PI: Three Latent Profiles

The five PI dimensions yielded low‐, moderate‐, and high‐PI profiles encompassing 30.4%, 43.4%, and 26.2% of participants. Entropy of 0.965 indicated that individuals were classified with high precision. Unlike a single total score, the profile solution preserved clinically relevant response‐pattern differences. Low scores were consistently observed in the low‐PI profile across domains like professional cognition, social support, social skills, managing career setbacks, and self‐reflection. Because previous nursing research links low PI with burnout and intention to leave [2, 4], this group may have the greatest need for support.

The moderate‐PI profile was largest. Its members appeared to have formed a professional role, although continued workplace support may be needed to maintain confidence. Scores in the high‐PI profile were elevated on every dimension, reflecting stronger professional values, constructive career reflection, and better perceived capacity to manage setbacks. The proportion with low PI was smaller than that reported in studies centered on novice nurses or students [2, 5]. A possible reason is career stage: established nurses have accumulated more clinical experience and organizational exposure than people beginning professional socialization. Prevalence estimates should therefore not be compared across populations without considering sample composition. Overall, the results demonstrate that PI is multidimensional and that support may need to be matched to profile‐specific needs.

### 4.2. Factors Associated With PI Profile Membership

Multinomial regression linked several demographic and work characteristics to profile membership. Nurses in management positions had greater odds of moderate or high PI. Such roles commonly provide more autonomy, involvement in decisions, contact with organizational leaders, and acknowledgment of contributions. These experiences may strengthen professional worth and align nurses’ career development with organizational priorities.

Income remained related to profile assignment after covariate adjustment, with higher‐paid nurses more likely to have moderate or high PI. Compensation may be interpreted as organizational recognition of nursing work [24]. We did not measure perceived pay equity, workload, or effort–reward balance, so the income findings indicate association rather than causation. Financial rewards may reinforce PI only alongside fair workload allocation, development opportunities, respect, and emotional support.

Alternatively, working in acute/critical care units and emergency departments correlated with a decreased probability of falling into the moderate‐PI and high‐PI groups. In these departments, nurses often encounter high patient acuity, time pressures, the threat of workplace violence, emotional challenges, and frequent exposure to critical situations [25]. All of which could erode professional confidence and diminish the internalization of positive roles. Earlier studies on emergency nurses indicate that workplace violence negatively impacts PI, but POS and resilience might mitigate this negative effect [13]. Hence, medical managers should not exclusively associate lower PI in these departments with personal coping mechanisms; Instead, they ought to enhance staffing, safety, debriefing, and support systems.

Interpretations of night‐shift findings should be approached with caution. After making adjustments, having more than 10 shifts per month did not significantly differentiate the high‐PI profile since its 95% confidence interval included 1; night‐shift frequency was not a trustworthy indicator in this particular sample. The likelihood of high PI was lower for women and nurses in secondary hospitals, but higher for those in specialized hospitals. The predominance of women and tertiary‐hospital staff in the sample necessitates confirmation through studies that have a more balanced gender distribution and include a wider range of hospital levels.

### 4.3. The Indirect Association Between CC and PI via POS

The analyses using correlation and SEM revealed positive estimates for CC–POS, CC–PI, and POS–PI, opposing the common assumption that compromise depletes resources and leads to negative outcomes. It should not be understood that making compromises in a career is beneficial or the same as actively adapting one’s career. Alternatively, the direction might reflect the wording and scoring of CC items and the overlap of constructs in self‐report instruments, common patterns in responses, the selective retention of nurses who remained in their jobs, or a different order of events than the model suggests.

Nurses who perceive career limits may attend more closely to how their organization responds through emotional concern, appraisal, advancement opportunities, and material resources. Theories of Social Exchange and Organizational Support indicate that perceived recognition and support can lead to a reciprocal increase in professional identification [10, 11]. Under Conservation of Resources Theory, POS is a contextual resource that may preserve nurses’ psychological resources while they adjust to career constraints [12]. The model therefore indicates covariation among adjustment, support, and PI, not a demonstrated sequence from resource loss through replenishment to identity. This reading is compatible with nursing evidence that POS relates positively to PI and can mediate or buffer associations involving stressful work conditions [13].

The substantial standardized CC ⟶ POS estimate (*β* = 0.901) might be due to conceptual overlap, common‐method variance, or a shared response style. As a result, the indirect effect should be viewed as tentative evidence of a connection, not as conclusive proof that CC elevates either POS or PI. Long‐term studies should investigate if compromise comes before shifts in POS and PI, and should evaluate different options, such as PI leading to CC and POS moderating the CC–PI link.

Managers might see a report of CC as an indication of employee issues and a prompt to review organizational support. The results suggest the need to assess support for nurses dealing with career constraints, but they do not prove that compromise strengthens PI. Without longitudinal evidence, any explanation remains speculative. Furthermore, the current CC tool primarily captures perceived deficits in opportunities, compatibility, and social norms. The unexpected positive CC–PI coefficient might be specific to the context, possibly due to measurement characteristics, common‐method effects, or reverse ordering. This interpretation should be tested using measures that differentiate between passive compromise and active adjustment.

### 4.4. Practical Implications and Intervention Suggestions

Based on the above findings, four profile‐specific and management‐oriented recommendations are proposed:

1. Adopt profile‐oriented interventions. Managers should focus on career counseling, mentorship, resilience training, and opportunities for professional reflection for nurses with low PI. For individuals in the moderate‐PI category, the focus should be on enhancing professional development, offering feedback, and avoiding a shift to the low‐PI profile. To uphold their role‐model status, nurses in the high‐PI group should receive leadership training and opportunities for professional growth.
2. Strengthen support for high‐risk departments. Nurses in acute/critical care units and emergency departments should receive targeted support, including adequate staffing, violence‐prevention systems, psychological debriefing after critical events, peer‐support programs, and protected rest time. Department‐level interventions should focus on improving both safety and professional recognition, rather than relying solely on individual resilience.
3. Build a multidimensional organizational support system. Given that POS partially mediated the CC–PI association, hospitals should provide both emotional and instrumental support. Emotional support may include respectful communication, recognition, supervisor care, and psychological services. Instrumental support should include fair promotion pathways, transparent performance appraisal, continuing education, workload optimization, and flexible scheduling.
4. Exercise caution when interpreting the data on income and night shifts. The association between higher income and membership in moderate‐PI and high‐PI categories suggests that fair wages could bolster professional recognition. After adjustments, the frequency of night shifts was not a strong indicator of high‐PI membership. Thus, managers should avoid using frequent night shifts as a sign of dedication. Alternatively, they should establish fair scheduling systems and monitor fatigue, sleep, and long‐term retention.

### 4.5. Limitations

There are several limitations to interpretation. Cross‐sectional data cannot establish the temporal direction of SEM paths. The reliance on convenience sampling in Henan Province, mainly from tertiary hospitals, hinders the ability to apply the results to different regions and to primary or secondary institutions. Given that all constructs were self‐reported, there is a chance of bias from common methods and socially desirable answers. Going against typical interpretation, the positive CC–PI estimate might reflect the measurement conditions, shared approach, or reversed order. Perceived losses in career chances and suitability are also stressed by the CC measure, which might not fully reflect active adaptation. There is a need for instruments that can tell passive compromise apart from active adjustment. Lastly, the three‐profile solution should be tested on an independent sample to verify its stability and clinical value.

Longitudinal or experimental work is needed to establish temporal ordering among CC, POS, and PI. Replication across geographic areas and hospital levels should assess whether the PI profiles generalize. Interviews could clarify how hospital nurses in China interpret CC. Future analyses should also compare competing SEM specifications, evaluate POS in mediator and moderator roles, and supplement questionnaires with organizational records of workload, staffing, advancement opportunities, and turnover.

## 5. Conclusion

We identified low‐, moderate‐, and high‐PI profiles. Income, management role, department, gender, hospital level, and hospital type differentiated membership, but night‐shift frequency did not differentiate high PI after adjustment. CC, POS, and PI were positively associated, with POS accounting for an indirect component of the CC–PI relationship. In this sample, the positive CC–PI coefficient may reflect pragmatic adjustment as well as perceived occupational constraint. Profile‐specific measures, stronger organizational support, and focused attention to acute/critical care and emergency nurses may help sustain PI.

## Funding

The authors have nothing to report.

## Ethics Statement

We obtained approval from the Ethics Review Committee of Zhengzhou University (Approval No.: ZDHL20250501). All participants in the study provided electronic informed consent. All participants volunteered to complete the questionnaires after being informed of the study purpose, and only those who agreed to participate were enrolled.

## Consent

Please see the Ethics Statement.

## Conflicts of Interest

The authors declare no conflicts of interest.

## Data Availability

The corresponding author can provide the datasets used and/or analyzed in this study upon reasonable request.
